# Endoscopic Retrograde Cholangiopancreatography in Pediatric Population: A Decade-Long Experience from 2 Tertiary Centers

**DOI:** 10.5152/tjg.2025.24462

**Published:** 2025-01-13

**Authors:** Ersin Batıbay, Osman Yüksekyayla, Mahmut Polat, İbrahim Bayhan, Mehmet Sevinç, Ahmet Dağ, Sezgin Barutçu, Osman Hakan Kocaman, Mehmet Emin Boleken, Cem Şimşek, Fırat Erkmen, Cumali Efe

**Affiliations:** 1 Department of Gastroenterology, Harran University Faculty of Medicine, Şanlıurfa, Türkiye; 2 Department of Gastroenterology, Mehmet Akif İnan Training and Research Hospital, Şanlıurfa, Türkiye; 3 Department of Internal Medicine, Harran University Faculty of Medicine, Şanlıurfa, Türkiye; 4 Department of Anestesiology, Harran University Faculty of Medicine, Şanlıurfa, Türkiye; 5 Department of Gastroenterology, Gaziantep University Faculty of Medicine, Gaziantep, Türkiye; 6 Department of Pediatric Surgery, Harran University Faculty of Medicine, Şanlıurfa, Türkiye; 7 Department of Gastroenterology, Hacettepe University Faculty of Medicine, Ankara, Türkiye; 8 Department of General Surgery, Balıklıgöl State Hospital, Şanlıurfa, Türkiye

**Keywords:** Biliary interventions, choledocholithiasis, endoscopic sphincterotomy, ERCP, hydatid cyst

## Abstract

**Background/Aims:**

Endoscopic retrograde cholangiopancreatography (ERCP) is an essential diagnostic and therapeutic method for pancreato-biliary disorders in adults, but its use in pediatric populations remains limited. This study aims to evaluate the indications, technical success, and safety of ERCP in pediatric patients.

**Materials and Methods:**

A retrospective analysis of all ERCP procedures performed on patients under 18 years of age was conducted at 2 tertiary centers in Türkiye (Harran University and Gaziantep University Hospital) during the period between January 2013 and May 2024. The data used for the study were obtained from patients’ medical records.

**Results:**

A total of 153 ERCP procedures were performed on 83 pediatric patients (64%, female) with a mean age of 12.9 years (range 3-17) at the time of ERCP. Common bile duct stones were the most frequent indication (n = 39, 47%) for ERCP, followed by biliary hydatid cyst-related complications (n = 24, 29%). The overall cannulation success rate was 98.7% (82/83). Endoscopic sphincterotomy was performed in 79 (95%) patients. More than one ERCP procedure was performed in 41 (49%) patients. Stones were extracted in 32 patients (30 were biliary and 2 pancreatic). Stent placement was performed in 33 patients (25 biliary and 8 pancreatic). Post-ERCP pancreatitis developed in 4.8% (n = 4) of patients, and all classified as mild. Ten (12%) patients developed mild/moderate cholangitis following ERCP. One patient (1.2%) experienced minor bleeding. About half of the patients (48%) were discharged within 1 day post-procedure. No patient experienced procedure-related mortality.

**Conclusion:**

Our study results indicate that ERCP is both effective and safe in the pediatric population.

**Main Points**

- Little is known about the efficacy and safety of ERCP in pediatric population.
- A large cohort of pediatric patients who underwent ERCP from 2 tertiary centers was presented.
- All procedures were performed by adult endoscopists.
- Our data suggest that ERCP is safe and effective in pediatric patients.

## Introduction

Endoscopic retrograde cholangiopancreatography (ERCP) has revolutionized the diagnosis and management of pancreato-biliary disorders in adults, offering a minimally invasive alternative to traditional surgical approaches.^1-4^ However, the application of ERCP in pediatric population presents unique challenges and considerations. The lower incidence of pancreato-biliary diseases in children, along with technical difficulties and anesthesia-related concerns, has limited the widespread adoption of ERCP in pediatric gastroenterology.^5,6^

Endoscopic retrograde cholangiopancreatography experience is limited in pediatric patients mainly due to the lower incidence of pancreato-biliary disorders in children than in adults. However, technological advancements and growing endoscopists’ expertise enabled the more common use of ERCP in pediatric patients, although the overall volume of cases is still lower than in adults setting.^7,8^ The indications for ERCP in children show wide variations according to the patient’s age or geographical location. Congenital anomalies such as choledochal cysts predominating in infants and pancreatic disorders may be more common reasons for ERCP than biliary diseases when compared to adults.^9^

The success rates and safety profile of ERCP procedures are closely linked to the endoscopist’s experience. Therefore, pediatric ERCP procedures are more often performed by experienced adult gastroenterologists.^10^ The use of adult duodenoscopes in children weighing over 10 kg and older than 12 months has become standard practice, facilitating the transfer of adult ERCP techniques to pediatric populations.^10,11^ This approach traces its roots to the first reported pediatric ERCP, which was successfully performed using an adult duodenoscope.^11,12^

In this study, the aim was to elucidate the spectrum of indications, diagnostic yield, therapeutic interventions, and complication rates by analyzing a substantial cohort of the pediatric ERCP population. The authors believe that their data contribute significantly to the existing knowledge base by presenting a decade-long experience of pediatric ERCP procedures from 2 tertiary centers in Türkiye.

## Materials and Methods

### Study Design and Patient Selection

This multicenter, retrospective cohort study analyzed pediatric patients who underwent ERCP procedures at the Gastroenterology Departments of Harran Medical Faculty and Gaziantep Medical Faculty between January 2013 and May 2024. The study protocol was approved by the Harran University ethics committee (approval number: 2024.HRU/24.10.33, date: August 8, 2024). Informed consent was waived due to the retrospective nature of this study.

### Data Collection and Management

A standardized electronic data extraction form was used to collect information from patients’ medical records. The following data were retrieved: patient demographics (age, sex, weight, height), clinical characteristics (presenting symptoms, comorbidities), laboratory findings (complete blood count, liver function tests, amylase, lipase), diagnoses, ERCP indications, procedural details (duration, cannulation success), adverse events, and length of hospital stay. Pre-procedural imaging modalities (ultrasonography, magnetic resonance cholangiopancreatography) were documented, along with their findings. Two trained researchers independently extracted the data (O.Y. and M.P), and any discrepancies were resolved by consensus or consultation with a senior gastroenterologist (C.E., S.B., and C.S). Inclusion criteria encompassed all pediatric patients who underwent ERCP for any indication during the study period. Exclusion criteria were age ≥ 18 years, incomplete medical records, and procedures performed primarily for research purposes.

### ERCP Procedure

All procedures were performed by experienced adult gastroenterologists, each with a minimum annual volume of 700 ERCP procedures. Sedo-analgesia was administered by anesthesiologists using a combination of propofol (initial bolus of 1-2 mg/kg, followed by continuous infusion at 4-12 mg/kg/h), ketamine (0.5-1 mg/kg as needed), or midazolam (0.05-0.1 mg/kg). Patients were positioned in either the left lateral decubitus or prone position based on the endoscopist’s preference and patient comfort.

An adult duodenoscope (ED-530XT8, Fujifilm Corporation, Tokyo, Japan) was used for all procedures. Standard 0.035-inch guidewires and 5.5-Fr sphincterotomes were employed for cannulation and sphincterotomy, respectively. Cannulation techniques, including wire-guided cannulation and precut sphincterotomy when necessary, were performed according to standard protocols. Therapeutic interventions such as stone extraction, stent placement, and balloon dilation were carried out as clinically indicated.

### Prophylaxis and Periprocedural Management

Post-ERCP pancreatitis (PEP) prophylaxis was administered to patients in accordance with the current guidelines at the time of the procedure (100 mg indomethacin suppository rectally 30 minutes before the procedure). Ringer’s lactate solution was administered intravenously at a rate of 1.5 mL/kg/h, starting 4 hours before the procedure and continuing for 8 hours post-procedure.

Antibiotic prophylaxis (Ceftriaxone 50 mg/kg, maximum 2 g) was given to patients with suspected biliary obstruction or a history of liver transplantation. Post-procedure, patients were monitored in gastroenterology, pediatrics, or pediatric surgery wards as appropriate. Pain scores, serum amylase, and lipase levels were assessed at 4-, 12-, or 24-hours post-procedure.

### Outcome Measures

The primary outcomes were the technical success rate (defined as successful cannulation of the desired duct) and the adverse event rate. Difficult cannulation (inability to achieve selective biliary cannulation by standard ERCP techniques within 10 minutes or up to 5 cannulation attempts) was defined according to a recommended consensus report.^13^ Adverse events were classified according to the lexicon proposed by Cotton et al^14^ Secondary outcomes included diagnostic yield, therapeutic success rate, and length of hospital stay.

### Statistical Analysis

Data were analyzed using SPSS version 24.0 (IBM Corp.; Armonk, NY, USA). Continuous variables were presented as means ± SD or medians (minimum-maximum). Categorical variables were expressed as frequencies and percentages.

## Results

### Patient Characteristics

We included 83 patients (64%, n = 53 female) with a mean age of 12.9 years (range: 3-17) at the time of the ERCP procedure. A total of 153 ERCP procedures were performed on these 83 pediatric patients. The age distribution showed adolescent predominance, with 55% of patients between 13 and 17 years old. The youngest patient was a 3-year-old child who underwent ERCP for choledocholithiasis. The general characteristics of study population before ERCP are presented in Table 1. Eight patients had comorbidities: 2 patients had hereditary spherocytosis, 1 had both sickle cell anemia and thalassemia major, and 5 had celiac disease. Prior to ERCP, ultrasonography (USG) was performed in 58 (70%) of the patients while 25 (30%) patients underwent both USG and magnetic resonance cholangiopancreatography.

All 153 ERCP procedures were performed under sedo-analgesia administered by qualified anesthesiologists. This approach proved highly successful, with only 1 patient experiencing respiratory depression during the procedure. No patient required endotracheal intubation.

The carbon dioxide (CO_2_) was used in 34 (22%) procedures while 119 (78%) procedures were performed on room air.

**Figure 1.**
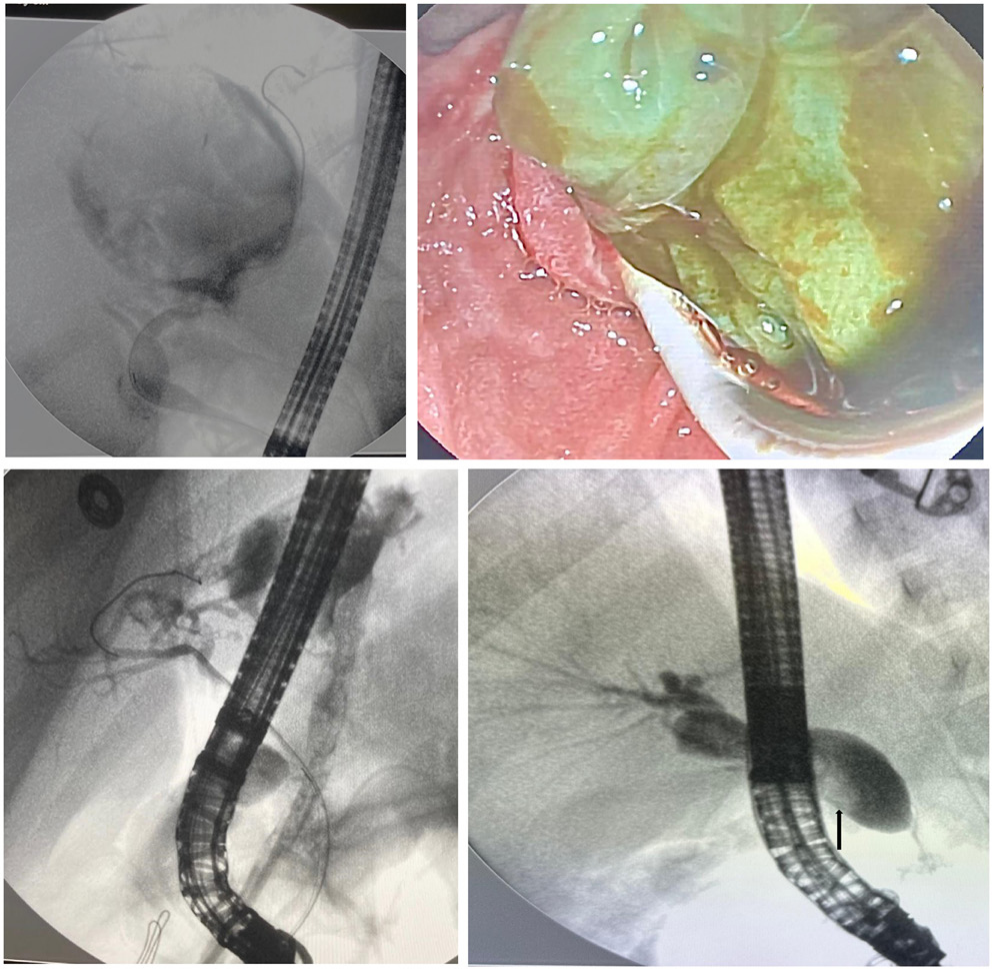
(A) A huge cystic lesion leading to compression and displacement of the major bile ducts in a 15-year-old boy. (B) Endoscopic retrograde cholangiopancreatography showing cyst membrane extraction from the bile ducts. (C) Post-operative cysto-biliary fistula in a 6-year-old girl. (D) Cholangiography during ERCP revealed filling defects in the common bile duct; about 10 mm diameter stone was removed.

Endoscopic retrograde cholangiopancreatography was performed due to hepato-biliary disorders in 75 (90.4%) patients, while 8 (9.6%) underwent ERCP for the management of pancreatic diseases. A total of 42 (50.6%) patients underwent a single ERCP, while multiple ERCP procedures were performed in 41 patients. Among hepato-biliary disorders, choledocholithiasis (n = 39) was the most common indication for ERCP, followed by hydatid cyst disease and its post-operative complications (n = 24), post-transplantation complications (n = 7), pancreatic diseases (n = 7), trauma (n = 5), and choledochal cyst (n = 1). Among the 5 patients who underwent ERCP for trauma-related complications, 1 had biliary duct injury (related to stabbing), and 4 patients had pancreatic duct injury (2 related to car/bicycle accidents, 1 related to suicide, and another related to stabbing). Figure 1 panel shows ERCP findings of hydatid cyst (A), removal of cyst membranes (B), post-operative cysto-biliary fistula (C), and stone in the common bile duct (D).

The technical success of our ERCP procedures showed a cannulation success rate of 99.3% (82/83). All therapeutic interventions and outcomes following ERCP are presented in Table 2. All cannulations were achieved by standard sphincterotome except that the needle-knife fistulotomy technique was performed as the first-line cannulation in 2 patients with papillary impacted stones. The data on cannulation duration was recorded in 42 patients, and the mean duration for cannulation was 6 minutes (range: 2-10). In 15 patients, the median number of cannulation attempts was 5 (1-8). Five (12%) patients met the criteria for difficult cannulation, including 3 post-operative cysto-biliary fistula cases, 1 patient with traumatic biliary injury, and other patient with choledocholithiasis. The data for the definition of difficult cannulation were not available for 26 patients. Selective cannulation failed in only 1 patient, who had mesenchymal tumor invasion of the common bile duct. One patient developed respiratory depression during ERCP (before cannulation was attempted). This patient recovered with supportive management (without intubation), and successful cannulation was achieved during the same ERCP procedure. No patient showed MRCP/ERCP features of pancreaticobiliary maljunction.

Endoscopic sphincterotomy was performed in 79 patients (95%) and the remaining 4 had prior ERCP with sphincterotomy. In 8 patients (9.6%), hydatid cyst membranes were successfully removed, highlighting the unique regional pathology encountered in our practice. Biliary stent placement was performed in 33 patients including complications of hydatid cyst (n = 18), post-transplant biliary stricture (n = 7), choledocholithiasis (n = 7), and traumatic biliary injury (n = 1). Pancreatic stenting was performed for post-traumatic complete disruption of the pancreatic duct (n = 4), pancreatolithiasis (n = 2), benign pancreatic stricture (n = 2), and prophylaxis against PEP (n = 1). For patients with post-traumatic pancreatic duct injury, 7-Fr 7 cm biliary stents without 2 side flaps were used for 2 patients; a 5-Fr 7 cm pancreatic stent was used for another patient, and for the remaining patient, sequentially 5-Fr and 7-Fr, 7cm stents were used. Biliary stents (7-Fr, 7 and 10 cm) without 2 side flaps were used for 2 patients with pancreatolithiasis. For 2 patients with benign pancreatic stricture, 5-Fr 10 cm pancreatic stents were used. A 5-Fr 5 cm pancreatic stent was used for a patient for prophylaxis of PEP.

### Adverse Events After Endoscopic Retrograde Cholangiopancreatography

Post-ERCP pancreatitis occurred in 4 (4.8%) patients (3 with hydatid cyst complications and 1 trauma-related liver injury). All cases were classified as mild, requiring only conservative management. One patient experienced minimal bleeding, which was easily controlled by epinephrine-saline solution injection therapy. Ten patients, including 6 with liver transplantation, 2 with choledocholithiasis, and 2 with hydatid cyst complications, developed cholangitis (mild, n = 8 and moderate, n = 2) following ERCP. No severe complications or procedure-related mortalities were observed.

Post-procedure recovery was generally good, with 48% of patients discharged following 1-2 days of ERCP. Two patients had the longest admission durations. One of them had multiple bone fractures following a car accident and was hospitalized for 25 days. Another patient had vertebral fractures and subsequently developed pneumothorax after a bicycle accident. This patient stayed 14 days in the hospital following ERCP for a pancreatic duct injury.

### Post-Endoscopic Retrograde Cholangiopancreatography Outcomes

The median duration of biliary stenting was 42 days (range: 14-380) in 33 patients. During 4-45 months of follow-up for patients who underwent ERCP due to hydatid cyst disease-related complications, 1 patient developed a biliary stricture that resolved after 3 ERCP procedures (balloon dilatation with stent placement). In transplanted patients, biliary stricture resolved in 4 patients, and all remained stent-free during 32-48 months follow-up, while recurrence of stricture developed in 3 patients who are still undergoing ERCP for stent replacement. The median duration of pancreatic stent replacement was 59 days (range: 27-210 days) in 8 patients who underwent ERCP for pancreatic disorders. During a median 19 months (range: 7-48 days) follow-up, 1 patient with pancreatolithiasis developed acute pancreatitis and was successfully managed conservatively. No patient developed features of pancreatic enzyme insufficiency or any other major complications.

## Discussion

Our study results contribute significantly to the existing literature on pediatric ERCP, given its substantial patient cohort and the number of procedures performed. The high volume of procedures in the centers likely contributed to the favorable outcomes observed, particularly the low complication rates, which are comparable to or lower than those reported in some previous studies. Overall, it was confirmed that ERCP is safe and effective in pediatric populations when it is performed by expert endoscopists.

The relative rarity of pancreato-biliary diseases in children, coupled with technical challenges and limited endoscopist expertise, has resulted in pediatric ERCP studies with small sample sizes.^15,16^ Our data were derived from 2 large-volume ERCP centers and involved endoscopists in this study who perform more than 700 cases of ERCP per year. Therefore, failed cannulation rates and the frequency of other adverse outcomes such as PEP or bleeding were very acceptable in our study.

In our study, patients’ ages (range: 3-18 years) are in line with definition of a “child” according to the World Health Organization. Some studies have extended their inclusion criteria to patients up to 21 years old.^17^ In one study,^18^ a total of 56 ERCP procedures were performed on patients who were younger than 1 year old and all weighed less than 10 kg. In this study, a pediatric duodenoscope was used for patients younger than 3 years old while another study^18^ reported 89% cannulation rates in the infant population. Our successful cannulation rate was 98%, which is in line with 2 other studies^6,8^ that reported 95% and 94% cannulation rates, while Åvitsland et al^18^ reported 89% cannulation rates in the infant population. These results suggest that age (< 3 years) seems to be an important predictor of successful cannulation in pediatric ERCP. The limited navigational space for the endoscope, quick development of small bowel gas distension that causes respiratory restriction during the procedure, even with the use of CO_2_, and the possibility of compression of the large vessels during endoscope insertion may explain the technical and procedural difficulties in infants (< 3 years) ERCP. It seems that a standard-sized (adult) duodenoscope is more likely to cause these adverse outcomes. Therefore, in children (< 3 years old or 10 kg), a pediatric duodenoscope is recommended, but pediatric duodenoscopes are not available in all endoscopy units, and their narrow instrument channel renders it difficult to perform some interventions.^19^

The indications for ERCP in pediatric patients often vary with age and are predominantly therapeutic, focusing on biliary pathologies. In infants and young children, congenital and anatomical abnormalities are the primary indications, while adolescents more frequently require ERCP for biliary obstructions.^8^ A study focusing on infants with a mean age of 10.4 months reported that 88.4% of procedures were therapeutic, with 47.1% related to pancreato-biliary anomalies.^20^ Another large-scale study involving 856 procedures in 626 patients across different age groups found that biliary atresia was the most common indication in infants (< 1 year old), and choledochal cysts and choledocholithiasis in children aged 1-6 years, and choledocholithiasis and pancreas pathologies in those aged 7-19 years.^21^ In our study, choledocholithiasis was the leading indication, followed by hydatid cyst disease. The high prevalence of hydatid cyst disease in our cohort is a notable finding that differs from much of the existing literature. This can be attributed to the geographical location of our centers in regions where hydatid disease is endemic.^22,23^

Overall, reported complication rates of ERCP in children have decreased over time. Similar to the adult population, rectal indomethacin and Ringer’s lactate infusion was administered to pediatric patients.^24^ This management may be the reason for decreased incidence of PEP, but we cannot definitively assess the efficacy of this prophylactic regimen due to the lack of a control group. Two studies^25,26^ from the 1990s reported 17% and 13% rates of PEP, and this rate was 9.7% in another later study.^27^ A more recent multicenter study^28^ no cases of PEP and only 1 minor bleeding among 126 pediatric patients who underwent ERCP. Finally, a large cohort-based review that included 7168 cases reported 4% PEP rates.^29^ A low PEP rate was reported (4.8%), which is consistent with recent literature. These results also suggest that improved technical procedures and endoscopists’ experience are the major factors in decreasing the frequency of PEP.

Bleeding and infections are other ERCP-related complications that have also reported in pediatric patients.^10^ In our study, only 1 (1.2%) patient developed mild bleeding. A recent systematic review^16^ reported 0.6% bleeding rates, which suggests that bleeding is a very rare complication in pediatric ERCP. Post-ERCP cholangitis was noted in 12% of our patients, which is higher than that reported in the systematic review (0.8%) of pediatric ERCP.^16^ These discrepancies can be explained by the characteristics of patient populations. In our study, 31 (37%) patients underwent ERCP for the management of biliary complications of liver transplantation and hydatid cyst. These patients are high-risk groups for the development of post-ERCP cholangitis. Overall, mortality rates were less than 0.2% in a national database analysis.^9^ Importantly, this study did not provide details of the patients who died, and it is difficult to know whether the cause of death was the underlying disorder or ERCP itself. In our population, no patients needed surgical intervention, and no mortality was observed. To our knowledge, mortality was not reported in other studies.^6-8,11,12^ Therefore, this specific report should not discourage physicians from performing ERCP in patients with appropriate indications.

Our study has some limitations. This study is retrospective, and it is not possible to collect all data from patients’ medical records. Endoscopic Retrograde Cholangiopancreatography indications show variations between Western and Eastern populations. Therefore, our results may not be generalizable to all populations. Detailed information about difficult cannulation in 26 patients could not be provided, but clear information about ERCP success rates in pediatric patients and all relevant adverse outcomes were provided. Therefore, these limitations do not introduce a major bias in our main findings.

In conclusion, the study’s results showed that ERCP is effective and safe in pediatric populations. In children, ERCP should be performed by experienced practitioners in well-equipped centers. These results will also be useful for establishing future comprehensive guidelines for pediatric ERCP that can further enhance the management of these complex cases.

## Figures and Tables

**Table 1.** General Characteristics of Study Population

Total patients	n = 83 (%)
Gender (female)	53 (63.8)
Age (years)	
0-5	3 (3.6)
6-12	34 (40.9)
13-17	46 (55.4)
Comorbidities	
Hereditary spherocytosis	2 (2.4)
Sickle cell anemia	1 (1.2)
Celiac disease	1 (1.2)
Thalassemia major	1 (1.2)
Cerebral palsy	1 (1.2)
Gordon syndrome	1 (1.2)
Polycystic kidney disease	1 (1.2)
Reasons for ERCP	
Choledocholithiasis	39 (46.9)
Hydatid cyst complications	24 (28.9)
Post-transplantation complications	7 (8.4)
Pancreatic diseases	7 (8.4)
Trauma	5 (6)
Choledochal cyst	1 (1.2)

**Table 2.** Therapeutic Interventions and Outcome after ERCP

Number of ERCP Procedures	Patients (%)
1	42 (50.6)
2	24 (28.9)
3	10 (12)
≥ 4	7 (8.4)
Median cannulation duration (minute)	6 (2-10)
Median cannulation attempts	5 (1-8)
Endoscopic sphincterotomy	79 (95)
Biliary stone extraction	30 (36.1)
Hydatid cyst membranes removal	8 (9.6)
Stricture dilation (ballon /bougie)	10 (12)
Biliary stent placement	33 (39.7)
Pancreatic stent placement	8 (9.6)
Pancreatic stone extraction	2 (2.4)
All complications	4 (1.2)
Pancreatitis (mild)	4 (4.8)
Bleeding (mild)	1 (1.2)
Infection/cholangitis (non-severe)	10 (12)
Discharge (days)	
1	13 (15.6)
2	26 (31.3)
3	24 (28.9)
≥ 4	20 (24.1)

## Data Availability

The data that support the findings of this study are available on request from the corresponding author.
